# Mental health problems and their related factors among seafarers: a scoping review

**DOI:** 10.1186/s12889-022-12713-z

**Published:** 2022-02-11

**Authors:** Woraluk Jonglertmontree, Orawan Kaewboonchoo, Ikuharu Morioka, Plernpit Boonyamalik

**Affiliations:** 1 Department of Public Health Nursing, Faculty of Public Health, Mahidol University, 420/1 Ratchawithi Road., Ratchathewi, 10400 Bangkok, Thailand; 2 Graduate School of Health and Nursing Science, Wakayama Medical University, Mikazura 580, 641-0011 Wakayama, Japan

**Keywords:** Mental health, Psychological issues, Factors, Seafarer, Maritime, Scoping review

## Abstract

**Background:**

Seafarers are often reported to be engaged in a dangerous physical and psychosocial work environment. However, mental health status among seafarers has not been focused on compared with physical health issues. Systematic, comprehensive reviews of mental health problems and their relevant factors are lacking. This review aimed to clarify beneficial approaches to the mental health problems faced among seafarers using a scoping review to systematically map the evidence regarding mental health issues and their related factors.

**Methods:**

Studies were searched on MEDLINE/PubMed, Science Direct, Academic search complete using EBSCOhost databases, SCOPUS, EMBASE, and Web of science on 20, August 2020. This scoping review was conducted based on the framework of Arksey and O’Malley and Preferred Reporting items for Scoping Reviews flow diagram. The inclusion criteria were studies which determined the relationship between factors relevant to working conditions or working environment, and mental health in seafarers, and etc. Data were narratively summarized and reported.

**Results:**

Twenty-four were included in this review while two major findings were clarified. Firstly, the prevalence of stress, depressive symptoms, and burnout have been mentioned for decades. Secondly, factors related to mental health and psychological issues can be categorized as individual and work environmental factors. The individual factors include experience, age, health status (high BMI, poor sleep, and diabetics), and resilience. The work environmental factors consist of two parts. Job demands comprise pressure from contractors/customers/time, working hours, ship department, job title, voyage episodes, period of seafaring, noise, and vibration. The job resources included instrumental support, team cohesion, shipboard caring and effort-reward imbalance.

**Conclusions:**

A beneficial approach to mental health problems faced among seafarers is necessary to understand comprehensively at individual and organization levels. Promoting health behaviors, training resilience, and managing obesity and chronic diseases comprise individual level strategies. Providing seafarers with adequate instrumental support, and practical support to communicate with customers, managing their distinct work-rest hours and adequate effort-reward balance comprise organization level methods.

## Introduction

Seafarers are often reported to be engaged in a dangerous physical and psychosocial work environment [1–3]. Risk factors against health conditions include heat, cold, noise and vibration, multiculturalism and multinationalism, social isolation and loneliness, separation from spouses and families, piracy, and criminalization on board [3–5]. Seafarer’s tasks are characterized by hierarchical work structure, shift work, and indistinct work-rest areas [6]. These job demands for seafarers impact on physical health, and psychological issues, such as turnover intention, job dissatisfaction levels, and in some cases cause a variety of mental health problems: stress, depression, burnout, and, at its worst, suicidal ideation [3, 7]. A related review of the suicidal rate demonstrated that 5.9% of seafarers died by suicide from 1960 to 2009 [3]. Mental health status among seafarers should be emphasized.

Mental health status among seafarers have not been focused on compared with physical health issues [2]. Studies on mental health status among seafarers are limited to the latest three review articles. The first one showed that the number of studies on psychological functioning and various aspects of mental health among maritime workers was low by study classification, accounting for only 10.61% of the total [8]. The second one reported that mental health status could be evaluated using the prevalence of suicide and missing at sea rates of seafarers, who are assumed to have committed suicide. Missing at sea cases might have resulted from personal factors and seafaring work environment, although this was unreferred to in the studies using an association or causal relationship study design [3]. The last one focused on risk of depression and suicide based on the evidence of stress and loneliness even when studies on the depression and suicide among seafarers were scarce and fragmented [7]. This indicates that systematic comprehensive reviews on mental health problems and their related factors are not available and scoping reviews are lacking in seafarer area. In 2012, the UK and Australia launched a valuable project related to mental health to optimize health status and well-being among seafarers [3].

Studies focusing on well-being or psychological aspects of health among seafarers have not been extensively conducted compared with research concerning physical health even though working conditions of seafarers are physically and psychosocially dangerous. Thus, the present review aimed to clarify beneficial approaches to mental health problems faced by seafarers using a scoping review to map systematically the evidence regarding mental health issues and their related factors.

## Methods

This scoping review was conducted based on the framework of Arksey and O’Malley methodological framework [9] and the Preferred Reporting items for Scoping Reviews flow diagram (PRISMA-ScR) [10].

This review was guided by two review questions: “what mental health problems or psychological issues are described in the literature topics among seafarers?” and “which factors are related to mental health problems among seafarers?”

### Searching strategy

Studies were searched using electronic databases: MEDLINE/PubMed, Science Direct, Academic search complete through EBSCOhost databases, SCOPUS, EMBASE, and Web of science to identify relevant published articles. Relevant research was searched 20 August 2020. Studies were limited to peer-reviewed, written in English and published from 2010 to 2020.

### Eligibility criteria

Research questions guided the searching terms and eligibility criteria. The determined inclusion and exclusion criteria are shown in Table 1. Studies meeting the inclusion criteria were eligible for review regardless of age, gender, race and country of the subjects. The studies included those conducted in term of observational studies, qualitative, mix methods and experimental research designs among workers in the maritime industry. However, reviews, letters, editorials, conference papers, policy statements and books were excluded. Full texts had to be published and available in English language.

**Table 1:** Inclusion and exclusion criteria

Inclusion criteria	Exclusion criteria
• Text was written in English • Study subject was a worker on ship, a worker at sea, a seafarer, a worker in the maritime industry, or a worker in a commercial fleet or merchant ship. • Outcome was mental health, psychological or psychosocial issues. • Articles determined the relationship between factors relevant to seafaring working conditions or working environment, and mental health or psychosocial issues.	• Study subject was in the navy with pre-postcombat/deployment and had worked on a royal navy ship. • Study subject was a worker in the oil and gas industry or an offshore-onshore worker. • Study subject experienced mental health illness or was under treatment for a mental health illness.

### Study selection

**Fig. 1:**
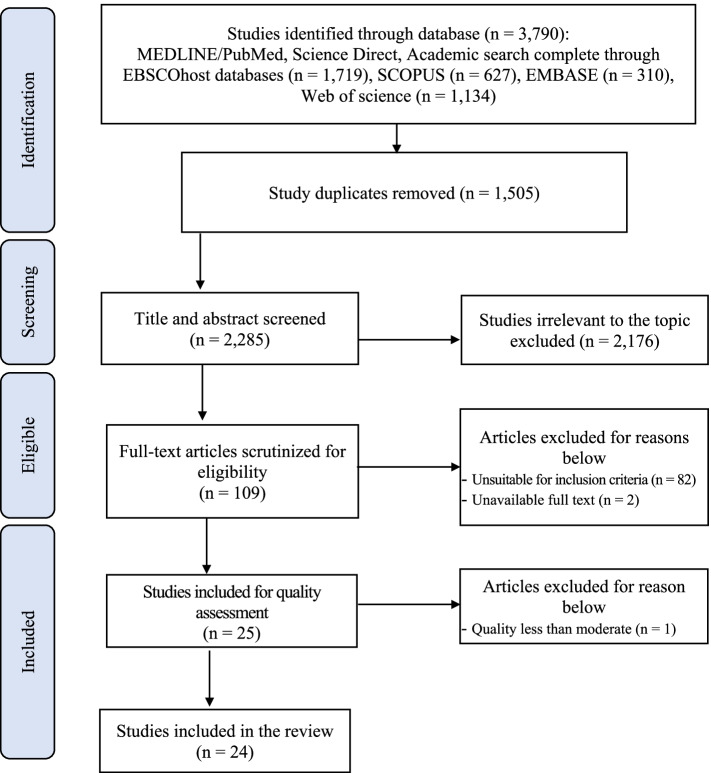
PRISMA-ScR flow diagram describing the process of study selection

All the identified studies were imported into an EndNote 20 [11]. After removing any duplicates, the predetermined eligibility criteria were applied to assess the identified studies using a two-step process. Firstly, title and abstract of studies were broadly screened to exclude studies that were obviously irrelevant to the topic of the present review by the two authors. Secondly, two authors independently scrutinized the full text of studies based on the inclusion and the exclusion criteria in Table 1. Whenever decisions of selected studies differed among the authors, issues were discussed until consensus was reached with the research team. The PRISMA-ScR flow diagram [10] describing the process of study selection is depicted in Fig. 1.

### Quality assessment

Although the phase on quality assessment was not discussed in the framework of Arksey and O’Malley [9], the methodologic quality in nonrandomized trials/studies were independently assessed by two reviewers applying the critical appraisal tool standardized by the Joanna Briggs Institute (JBI). This means cross-sectional studies (8 criteria) were categorized concerning the quality of the article as high (≥ 7 score), moderate (5-6 score), and low (< 5 score) [12]. Qualitative studies (10 criteria) were categorized as high (≥ 9 score), moderate (7-8 score), and low (<7 score) [13]. Studies exhibiting less than moderate quality were eliminated from the present review.

### Charting the data

Included studies were reviewed for characterizing general information: authors, publication year, country, study design, data collection, number of subjects, indicators, mental health problems, and related factors. They were charted int a Microsoft Excel database by the first author and verified by the other authors.

### Collating, summarizing, and reporting the results

The characteristics charted in the Microsoft Excel database were narratively summarized. Type of study, mental health or psychological issues, statistically significant factors in quantitative studies and all factors in qualitative studies were classified in domains for reporting and discussing.

## Results

### Searching and selecting the studies

A total of 3,790 studies were identified through the electronic databases. Of these, 1,505 were excluded because of duplicating. The titles and abstracts of 2,285 studies were screened to ensure they met the inclusion criteria. Next, 109 full texts were assessed for eligibility. Finally, 25 studies were selected for quality assessment.

Twelve studies possessed high quality using the critical appraisal tool, and 12 exhibited moderate quality. One study showing lower than moderate quality was excluded. Consequently, 24 studies were included in the present review (Table 2).

**Table 2:** Characteristics of included studies

Author (year)	Region (Country)	Study design	Data collection	Number of subjects	Indicator	Main finding	JBI Score
Chung et al. (2017) [6]	Asia (Korea)	Cross-sectional study	Self-administered questionnaire by sending questionnaire to captain, requesting him to notify seafarers onboard	160 seafarers container ship	Copenhagen burnout inventory (CBI)	Mean of personal burnout and work- related burnout were 35.70 and 26.29, respectively. Effort reward imbalance and sleepiness had positive effect on personal and work-related burnout. Work-related burnout mediated an incident at sea.	8
Oldenburg & Jensen (2019) [14]	Europe (Germany)	Cross-sectional study	Taking saliva sample	304 seafarers international container ship	Stress evaluated by cortisol level	Mean of stress was 0.308 (µg/dl). The highest value was among deck officers, followed by deck rating and engine room. The highest one during stay at port, followed by sea passage and river passage.	6
Haka et al. (2011) [15]	Europe (Denmark)	Cross-sectional study	Self-administered questionnaire by letter	346 seafarers	Item of perceived stress and isolation/loneliness	Stress and isolation/loneliness were 28.3% and 30%, respectively and demotivated going seafaring.	6
Doyle et al. (2016) [16]	Europe (Ireland)	Cross-sectional study	Ship’s web-base to upload online administered questionnaire	387 merchant seafarers (on board between 0 and 24 weeks)	Perceive stress scale assessment (PSS-4)	Mean of perceive stress among total subjects, East Asian, Caucasian, Latino/Hispanic, and South Asian were 1.32, 1.48, 1.11, 1.42, and 1.35, respectively. Higher level of resilience, longer seafaring experience and greater instrumental work support were associated with lower stress at sea.	8
McVeigh et al. (2019) [17]	Europe (Ireland)	Secondary data analysis	Ship’s web-based servers uploading online administered questionnaire	781 merchant seafarers	Perceive stress scale assessment (PSS-4) and job satisfaction scale	Mean of perceive stress and job satisfaction were 1.36 and 3.71, respectively. Dispositional resilience and higher instrumental support were associated with low perceive stress and high job satisfaction levels. Ratings and caterers as a non-officer job title had higher job satisfaction levels than officer of deck and engine division.	6
Oldenburg et al. (2013) [18]	Europe (Germany)	Cross-sectional study	A survey questionnaire with seafarers who attended a health examination before seafaring	251 seafarers on varied ships	Emotional exhaustion (EE) of the Maslach burnout inventory and the Epworth sleepiness scale	Burnout among total subjects, officers, ratings, and caterer were 10.8%, 10.7%, 4.5%, and 25%, respectively. Long working day (>10 h), insufficient sleep on board, lack of care taken by the shipboard superior and/or the shipping company, high responsibility for work organization, social problem due to long periods of separation from their family were associated with burnout.	7
Jurišić-Eržen et al. (2011) [19]	Europe (Croatia)	Cross-sectional study	Semi-structured interview, patient medical records, self-administered questionnaire	52 seafarers with type 2 diabetes and 56 non-diabetic seafarers	The Beck depression Inventory (BDI) and State-Trait Anxiety Inventory	Depressive symptom and anxiety were 34% and 46% among type 2 diabetics seafarers. Depressive symptom and anxiety were 11% and 20% among non-type 2 diabetics seafarers. Duration of shipping routes over 6 months, degree of BMI, glycemic control (HbA1c> 8%), and duration of diabetes were associated with depression.	8
Sąlyga & Kušleikaitė M (2011) [20]	Europe (Lithuania)	Cross-sectional study	A survey questionnaire from seafarers who attended a health examination before seafaring	1,930 seafarers	Subjective experienced psycho-emotional strain and fatigue experienced item	Duration of voyage after 2.6 months, higher age at 35-54 years, long working hour (9 – 12 h), higher education level, vibration and noise level, increased eyestrain, insomnia, depress, waist pain, and spinal pain were associated with psycho-emotional strain. Fatigue experience during 1-2 voyage (26%) was more than during 5 voyages (14%). Insomnia was associated with fatigue.	6
Nielsen et al. (2013) [21]	Europe (Norway)	Cross-sectional study	Questionnaire distribution while working on board	541 seafarers from 2 Norwegian shipping companies	Intention to leave and job satisfaction scale	Mean of intention to leave was 2.23. Age, individual intention and motivation to follow safety regulations, and team cohesion were negatively associated with intention to leave. Management prioritization of production over safety and high level of quantitative job demands (degree of difficult working conditions, pressure from customers/ contractor and stress related at work) were positively associated with intention to leave. Mean of job satisfaction levels was 4.17. Individual intention and motivation to follow safety regulations, laissez-faire leadership, and team cohesion were positively associated with job satisfaction. Management prioritization of production over safety and quantitative job demands (degree of difficult working conditions, pressure from customers/ contractor and job stress) were negatively associated with job satisfaction.	6
Bergheim et al. (2015) [22]	Europe (Norway)	Cross-sectional study	Questionnaire distributed when working on board	1,080 seafarers	Job satisfaction scale	Mean of job satisfaction levels was 4.17. Psychological capital (self-efficacy, optimism, hope, and resiliency) was related to job satisfaction.	8
Tedesco et al. (2018) [23]	Europe (Italy)	Cross-sectional study	Self-administered questionnaire by envelope	801 seafarers	Karasek Demand-Control-Support questionnaire	Mean of job demand and job decision latitude was 32.8 and 65.5, respectively. Job title as ratings, higher number of ports landed in a week, seafarers on cargo ship, and younger workers related to low job decision latitude.	6
Slišković & Penezić (2016) [24]	Europe (Croatia)	Quantitative: descriptive study	Online questionnaire (Electronic mail)	298 seafarers	5 items, overall and life satisfaction, 5-item mental health inventory (5 domains: anxiety, general positive effect, depression, behavioral control, and emotional control)	Mean of job, life satisfaction and overall mental health were 16.62, 15.23 and 22.5, respectively. Characteristic of employment contract including period on board between 2-4 months, favorable ratio between working and free days, and regular shift work were associated with job and life satisfaction. Duration onboard during 2- 4 months and regular shifts contract employment were related to good mental health.	7
Pauksztat (2017) [25]	Europe (England)	Qualitative study	Interview	54 seafarers cargo ship	N/A	Job demand was characterized as circadian disturbance from nigh work; inability to plan from unpredictable work schedule; workload from number of ports, days at sea, time in ports, collaboration with company, amount of cargo, paper work and planning, rule and regulations; difficulty from type of cargo, pilotage, maneuvering in ports, getting supplies, traffic, weather and seasons; and intrusions from collaboration in ports, pilot on board, and insecurity. These effect on working climate, fatigue, and turnover intention.	7
Tavacioğlu et al. (2019) [26]	Europe (Turkey)	Descriptive study	Face to face and online questionnaire	203 seafarers	Maslach Burnout Inventory (MBI) and Minnesota Job Satisfaction Scale (MJSS)	Mean of burn out and job satisfaction among total subjects were1.59 and 3.44, respectively. Seafarers at deck department had higher burnout score and lower job satisfaction score than those at engine department.	8
Oldenburg & Jensen (2019) [27]	Europe (Germany)	Cross-sectional study	Self-administered questionnaire and biometrically surveyed (physical activity and heart rate armband monitor) during working on board	323 seafarers container ship international seafarers	Subjective physical and mental work environment stress and strain SenseWear® armband monitor and Polar RS800	Mental and physical stress at port stay, river passage and sea passage (voyage episodes) were 37.8%, 24.8%, and 13%, respectively. Noise and vibration were a subjective strain but not objective strain. Distinction among voyage episodes had differently stress and strain.	8
Oldenburg & Jensen (2019) [28]	Europe (Germany)	Cross-sectional study	Self-administered questionnaire and biometrically surveyed (physical activity and heart rate armband monitor) while working on board	323 seafarers international container ship	Subjective physical and mental work environment stress and strain SenseWear® armband monitor and Polar RS800	Physical or mental stress among total subjects was 65%. Physical stress among deck rating department and engine department were 74.7% and 72.4%. Mental stress among deck officer department was 86.6%. Department in ship (deck officer, desk rating, and engine) had differently stress and strain.	7
McVeigh & MacLachlan (2019) [29]	Europe (Ireland)	Qualitative study	Focus group	32 Filipino merchant seafarers	20-item semi-structure focus group guidelines	Workload (stress and commercial pressure, and rest), safety, social, support, salary, food, shore leave and signing off and on, nationality and culture, management, inequality, and optimization were 11 domains related to experience of stress, resilience, and well-being.	8
Xiao et al. (2017) [30]	Asia (PR China)	Cross-sectional study	Self-administered questionnaire by training captains of the ship to administer questionnaire	917 seafarers at ports	Zung Self Rating Depression Scale (SDS)	Depressive symptom was 49%. Work-related psychosocial stress refers to worry about family member (66.5%), financial situation (55.4%), ship safety (64.3%), sea piracy (59.5%), port state control (51.5%), and occupational strain (52.7%).	8
Gu *et a*l. (2020) [31]	Asia (PR China)	Cross-sectional study	Self-administered questionnaire by online questionnaire	294 seafarers unspecified ship	Turnover intention by Mobley et al. and job demand by Karasek	Job demand consisting of job stress, time pressure and so on was positively predicted turnover intention (intention to leave).	5
Xia et al. (2016) [32]	Asia (PR China)	Cross-sectional study	Self-administered questionnaire, venous blood for neuroendocrine hormone level and menstruation record by training workers of the ship to collect the data	71 female seafarers in hospital ships	90-items self-rating psychological symptom scale (10 sub-scale such as somatization, obsessive-compulsive, anxiety, depression etc.)	Psychological evaluation of anxiety somatization, paranoia, and others were significantly high within 2-3 months of voyage. And psychological stress influences a body function including neuro-endocrine and immunological function during a long voyage.	6
Yuen et al. (2018) [33]	Asia (Singapore)	Cross-sectional study	Online questionnaire via electronic mail	116 seafarers	2-item job satisfaction survey	Rewards (high salary, family benefits, training opportunities, and promotion prospect), job stress, dispositional affect (positively affect, internal locus of control, and low burnout tendencies) and job characteristics (skill variety, task identity, task significance, autonomy, and job feedback) were related to job satisfaction.	7
Lefkowitz et al. (2019) [34]	North America (United States of America)	Secondary descriptive study	Data from large international marine insurance from 2007–2015	278 international seafarers	N/A	Mental illness including social withdrawal, mood swings or other concerning change was found 3.9 per 100,000 person-years.	6
Lefkowitz et al. (2020) [35]	North America (United States of America)	Cross-sectional study	Self-administered questionnaire at piloting training center	233 domestic seafarers	PHQ-9	Depressive symptom was 16%. Obesity (BMI > 35 kg/m^2^), poor sleep quality, and anxiety were associated with depressive symptom.	7
Silva et al. (2017) [36]	South America (Brazil)	Cross-sectional study	Self-administered questionnaire	316 seafarers in water transport company	20-items self-reporting questionnaire	Depressive/anxious mood was 14.5%. Female, family income, weekly working hours, self-report stress, thinking about quitting jobs, engine job, sedentary lifestyle, and not smoking were related to common mental health disorder.	5

### General characteristics of included studies

The general characteristics of 24 studies are demonstrated in Table 2. Sixteen studies were conducted in Europe [14–29], five in Asia [6, 30–33] two in North America [34, 35] and one in South America [36]. Twenty-two studies were quantitative [6, 14–24, 26–28, 30–36] and two were qualitative [25, 29].

Regarding study design, many applied cross-sectional designs. As to the data collection, five quantitative studies collected data using face to face questionnaires (two study locations were unidentified [19, 36], one study used a piloting training center [35], and two studies were conducted at a health examination hospital [18, 20]). Five studies distributed questionnaires using online surveys [16, 17, 24, 31, 33]. Five studies distributed questionnaires on board [6, 21, 22, 27, 28]. Two studies trained the captain and crews to serve as a co-researcher for collecting data on board [30, 32]. Two studies included subjects obtained by distributing questionnaire sheets using letters [15, 23]. One study’s data were collected using both face to face administered questionnaires and online method [26]. Four studies used biometrically surveys. (one study determined cortisol level in saliva [14], two studies used physical activity and heart rate armband monitors [27, 28], and one study used venous blood to assess hormone levels [32]). Two studies used secondary seafarer’s health data [19, 34], and one study used semi structured interviews cooperating with secondary health data and self-administered questionnaires [19]. Two qualitative studies applied focus group and interviews with seafarers [25, 29].

The number of subjects of quantitative studies ranged from 52 to 1,930. The participants of one study comprised female Chinese seafarers [32]. The others did not specifically identify sex. One study recruited seafarers with or without type 2 diabetics [19].

### Mental health problems and psychological issues among seafarers

Mental health problems and psychological issues among seafarers were explored in 18 quantitative studies. The incidence of mental illness among international seafarers, such as mood swing, and social withdrawal, totaled 3.9 per 100,000 person-years [34].

Stress among seafarers was reported in seven studies [14–17, 27, 28, 30]. Two studies revealed seafarers had perceived stress related to physical and mental work environment [27, 28]. One study showed that 65% of total subjects were stressed, that deck officers were the most mentally stressed and that deck rating personnel were the most physically stressed [28]. Another reported that 37.8% of seafarers during port stay, defined as after docking at the port until the departure, experienced physical and mental stress, and that this proportion was larger than those of seafarers involving river and sea passages [27]. Cortisol levels in saliva were the highest among deck officers, followed by deck ratings and engine room personnel, and was highest among seafarers on duty during port stay [14]. One study pointed out that 51.5 to 66.5% of seafarers experienced work-related psychosocial stress reporting worries about family members, financial situation, ship safety, sea piracy, port state control and occupational strain [30]. Similarly, one study reported that 28.3% of seafarers exhibited stress that demotivated seagoing [15]. In addition, two studies reported similar perceived stress scores [16, 17]. One study showed that stress differed by ethnicity on an international ship. East Asian seafarers had higher perceived stress scores than another ethnic seafarers, such as Caucasian, Latino/Hispanic and South Asian seafarers [16].

Burnout syndrome among seafarers was reported in two studies [6, 18]. The prevalence of burnout syndrome was 10.8% which differed among seafarer’s rank and job: officers rank (10.7%), ratings (4.5%), and galley staff (25%) [18]. However, one study demonstrated that the burnout score of personal issues was higher than that of work-related issues [6].

Depressive symptoms, anxiety, and loneliness and isolation were reported in five studies [15, 19, 30, 35, 36]. Three studies showed 14.5 to 49% of seafarers reported depressive symptoms [30, 35, 36]. Furthermore, 34 and 46% of seafarers with type 2 diabetes also reported depressive symptoms and anxiety, respectively [19]. One study showed that 30% of Danish seafarers felt isolation or loneliness [15].

### Factors related to mental health and psychological issues among seafarers

The factors related to mental health and psychological issues among seafarers were divided in two: individual and work environmental factors. Work environmental factors were based on the Job Demand-Resources Model [37].

### Individual factors

Experience of seafaring and age were referred to four studies [16, 20, 21, 23]. Those experiencing longer seafaring correlated with lower stress at sea [16]. Those at greater age were associated with psycho-emotional strain [20], but negatively associated with intention to leave [21], while younger age was a factor related to low decision latitude [23].

Health status was documented in four studies [18–20, 35]. Poor sleep quality or insomnia predicted psycho-emotional strain, fatigue [20], burnout [18], and depressive symptoms [35]. Those presenting type 2 diabetics were more than twice as likely to exhibit depressive symptoms and anxiety [19]. High BMI also was positively related to depressive symptoms among both general seafarers [35] and presenting type 2 diabetes [19].

Dispositional resilience and psychological capital work for positive psychology were represented as a protective factor in three studies [16, 17, 22]. Dispositional resilience was associated with lower stress [16, 17] and high job satisfaction levels [17], and psychological capital including resiliency was related to job satisfaction levels [22].

### Work environmental factors

#### Job demands

Pressure from contractors/customers/time and job stress in seafaring were negatively associated with job satisfaction levels [21] and positively associated with intention to leave [21, 31].

Long working stretches, more than 9 h daily, were related to psycho-emotional strain [20] and burnout [18].

Department on the ship was shown in two studies [26, 28]. One study demonstrated different mental and physical stress: the deck officer department introduced more mental stress, and deck ratings and engine personnel departments led to more physical stress [28]. Another showed that department on the ship predicted job satisfaction levels and burn out. Those in the deck department experienced higher burnout and lower job satisfaction levels than those in the engine department [26].

Job title was shown to be associated with psychological issues in two studies [17, 23]. Job title was classified in two groups: (1) officers such as captains and engineers and (2) nonofficers such as ratings or crew, and caterers. Having a lower job title was related to job decision latitude [23]. Ratings and caterers had higher job satisfaction levels than officers of deck and engine divisions [17].

Voyage episodes indicated different stress. Staying during port introduced more mental stress than that in river passage and sea passages [27]. The number of ports landed in weekly was related to job decision latitude [23].

Seafaring duration comprised a specific job demand in the maritime field. Short periods between - two to four months, favorable ratio between working and free days, and regular shifts were associated with high job and life satisfaction levels [24]. Seafaring after 2.6 months from the voyage start was more likely to produce psycho-emotional strain [20]. Among female Chinese seafarers, seafaring - two to three months introduced mental health problems, such as anxiety, somatization [32]. Seafaring over six months was related to depressive symptoms among seafarers with type 2 diabetes [19].

Ship noise and vibration, meaning a physical environment, were a subjective strain in the seafaring field in two studies [20, 27]. However, they had no effect on objective strain (heart rate and energy expenditure of physical activity) [27].

Two qualitative studies [25, 29] demonstrated the job demands identified through interviewing and collecting focus group data. Job demand included workload (stress and commercial pressure, and rest), shore leave, signing off and so on [29]. Job demand was characterized as circadian disturbance, workload, difficulty of work and work intrusions, introduced fatigue, poor working climate and increased turnover intention [25].

#### Job resources

Higher instrumental work support was a predictor of high job satisfaction levels [17] and low perceived stress at sea [16, 17].

Only one study reported team cohesion were a predictor of high job satisfaction levels [21].

A shipboard caring including laissez-faire leadership style were predictors of high job satisfaction levels [21]. Lack of care by a shipboard superior and/or a shipping company were related to burnout [18].

Effort-reward imbalance were related to burnout [6]. Reward (high salary, family benefits, and training opportunities) were predictors of high job satisfaction levels [33].

## Discussion

This up-to-date scoping review systematically mapped the results of studies examining seafarer’s mental health or psychological issues and their related factors over the past decade. This review included 24 studies representing 92% and 8% using quantitative and qualitative research designs. Regarding study design, all included studies employed a cross-sectional design. This highlights the need for further research using designs such as longitudinal study, case-control, or cohort study. As to collecting data, various methods were used, such as using face to face methods, surveying online, distributing questionnaire on board etc. This may have entailed that collecting data was difficult.

The current review highlighted the prevalence of mental health problems and psychological issues: stress ranged from 28 to 65% [15, 28]; depressive symptoms from 14 to 49% [30, 35, 36]; and burn out at 10.8% [18] during the past decade. However, the prevalence varied in a wide range. This may be explained from the heterogeneity of studies, use of different instruments, time frames, data collection methods, and multiple nationalities of seafarers.

To our knowledge, this review illustrated the wide range of individual and work environmental factors related to mental health and psychological issues.

Among individual factors, longer seafaring experience was one of the protective factors [16], and greater age increased psycho-emotional strain [20]. According to a related study, aid workers in aid organizations such as humanitarian assistance, and nongovernmental organizations showed older age and longer work experience played important roles as protective factors of mental health outcomes. Work experience corresponded to, but older age contrasted with the present study results. However, this should be compared cautiously because populations differ. Younger age was related to low decision latitude [23]. This result was supported by similar studies reporting age was associated with decision latitude among general practitioners in a community setting [38] and associated with depression among navy personnel assigned to an active-duty ship [39].

Poor sleep or insomnia exhibited a relationship with psycho-emotional strain, burnout, and depressive symptoms [18, 20, 35]. Seafarers have the possibility to sacrifice their sleep duration to catch up on work even as poor sleep habits occur among doctors, nurses, emergency services providers, gasoline station attendants, truck drivers, and others working 24-hour shifts [40]. This was supported by the related longitudinal studies and systematic reviews showing that poor sleep and insomnia produced depression at least twice the number of times as those of young and elderly men patients with insomnia [41–44].

Health status, high BMI and type 2 diabetes were risk factors for depressive symptoms among seafarers [19, 35]. High BMI showed a strong association with depressive symptoms in adolescent and worker age group [45, 46]. Overweight status may influence bodily inflammation preceding depression [46]. In general, the risk of developing depression is increased nearly twice among people with diabetes, but the linkage between them remains unclear. Biochemical changes such as arousal of the nervous system, could account for an increased risk of depression among individuals with diabetes [47], although glycemic control (HbA1c) and average blood glucose level showed no significant association with depression [48]. These results suggested that seafarers with high BMI and type 2 diabetics should be screened for depression while onboard.

Dispositional resilience plays a role in protective factors among seafarers [16, 17]. Resilience works as a personal resource of resistant stress, growth, and personal development that may foster the ability to cope with the environment [49] among general adults [50] as well as active duty mariners [51]. Resilience is also a resource of coping with job demand and development of job resilience [52]. Thus, resilience is also effective for seafarers to prevent mental health problems. Since resilience is improved by training [53], it should be therefore suggested to be included in a seafarer’s pre-onboard program.

Among the job demands in work environmental factors, over 9 h of daily work introduced psycho-emotional strain [20] and burnout [18] among seafarers. Similarly, among those working over 40 h weekly and averaging more than 8 h daily reported negative impacts with mental health consisting of burnout [54], depressive symptoms [55–57], stress, suicidal ideation [55] and well-being [56].

Deck department and engine department are the two main departments on a ship. In general, deck officers are responsible for paperwork similar to white collar workers, and ratings and engine personnel are responsible for engine work similar to blue collar workers. In this review, deck officers experienced more mental stress, a higher possibility of burnout, and lower satisfaction levels than ratings and engine personnel, while ratings and engine personnel were more physically stressed than deck officers [28]. This was contrary to the results of the study showing that blue collar workers had higher perceived stress than white collar workers [58]. Deck officers carry out not only paperwork but also commanding tasks with high work responsibility at remote areas contributing to their higher stress levels than those of general white collar workers.

Job titles in seafarer’s work are classified as officers at a higher rank and nonofficers at a lower rank. The commanding system is based on the work hierarchy concurring with military work. Two earlier military studies reported that lower rank was associated with psychological issues [59, 60]. This study revealed that the lower ranked nonofficers, called ratings or crews and caterers, had higher job satisfaction levels than those of officers in deck and engine departments [17]. This fact did not correspond with those two studies, so evidence may support that the hierarchy command environment in spite of being on a military or merchant ship was associated with psychological issues.

The ship voyage, a unique seafarer’s work condition, consists of port stay, sea passage, and river passage. The result in this review showed that port stay introduced more stress than other episodes [27]. Different tasks of each episode require different demands. Port stay is physically demanding: loading and unloading operations, working as a watch keeper, repairing an engine, refueling, and intaking provision; and psychosocial demanding: requiring contractors or customers [61].

Long seafaring periods can induce mental health problems. This result was consistent with earlier studies that the navy crew during shipboard deployment were more likely to become depressed than those before and after three months deployment [39], and that work environments, involving living alone, were risk factors for depression [62]. The seafarers work and living conditions with limited numbers of people in a limited area for long consecutive periods may cause seafarers mental health problems particularly depression.

Noise constituted a risk in a review of seafaring occupations published in 2010 [4]. Noise still showed a relationship with subjective strain in this review. A related study in a large working population [63] and a longitudinal study [64] also showed strong associations between personal perception of noise level related to depressive symptoms and suicidal ideation. Vibration was in accordance with earlier empirical evidence that whole body-vibration at 3-20 Hz frequency increased mental demand [65].

Job demands in seafaring may have their own specific factors as specific job demands in each occupation cause psychological problems [66]. Psychosocial environment factors including pressure from contractors/customers/time, and poor working environment [21] were directly related to psychological issues.

As to job resources in work environmental factors, instrument support, shipboard caregiving, team cohesion and reward were related to high job satisfaction levels [17, 21, 33]. On the contrary, lack of caregiving and effort-reward imbalance related to burn out [6, 18]. These results were supported by the evidence empirically demonstrating that neither supportive coworkers nor supervisors present had the possibility to increase mental distress in an offshore petroleum industry [67, 68]. Also, the results corresponded to an earlier systematic review with meta-analysis, showing that effort-reward imbalance, high job demand, and low social support were positively associated with mental health disorders, and served as risk factors [69].

### Beneficial approaches to mental health problems among seafarers

Beneficial approaches could be derived at individual and organization levels. At the individual level, promoting health behaviors such as healthy diet consumption and physical exercise on the ship is highlighted. Providing information on healthy daily meals using a cooking course and improving fitness facilities are preferred to be provided by the shipping company manager. This allowed seafarers easy access to health behaviors [70]. Training resilience is also important. For this purpose, physical exercise constitutes one specific method including programs combining exercise with resilience training [71, 72]. In particular, promoting health behaviors and developing resilience are emphasized for inexperienced seafarers, young and low ranking seafarers, and those working during port stays. Health management is also needed because obesity and diabetes have been associated with mental health problems. Regular clinical tests for seafarers are required to detect such disorders.

At organization level, a company manager provides seafarers with sufficient instruments, such as quality noise protective equipment because noise from engine is a subjective strain. While on board, seafarers experience high pressure from contractors or customers due to restricted work time. Facilitating practical support for better communication among them is preferred. Seafarers work in unclear work-rest cycles for long hours. Even with the difficulty of securing a safe break-rest location, providing a proper break time is desirable. Effort-reward imbalance constitutes a major source of stress response. This reward includes not only financial but also psychological and career rewards. Lastly, establishing an appropriate promotion system is desired.

#### Strengths and limitations

This scoping review followed the framework of Arksey and O’Malley [9], the PRISMA-ScR flow diagram [10], and the determined eligibility criteria. Because no restrictions were placed on the study type, a variety of research designs, such as quantitative, qualitative, or mixed-method were included. Then a quality assessment was conducted for the included studies using the standardized critical appraisal tool standardized by JBI [12, 13].

Limitations were encountered concerning study design. First, almost all included studies used the design of a quantitative cross-sectional survey. This is possibly a common method, but a causal relationship between mental health problems and investigated factors needs to be cautiously interpreted. Second, the included studies used self-report and may have resulted in self-reporting bias. Only two studies analyzed the measured outcome of objective parameters. Third, the characteristics of the sampled subjects is biased. Thus, indicating mental health problems among all seafarers would be difficult because job departments or shipping routes were undistinguished such as deck officers, non-officers, and engine personnel, sea or river passage, and types of ship. These factors were likely to introduce different mental health disorders. This highlights the need of further research in the maritime field.

## Conclusions

This scoping review notes that the prevalence of stress, depressive symptoms, and burnout have been mentioned for decades. The factors related to mental health and psychological issues can be categorized as individual and work environmental factors. These results comprehensively offer beneficial approaches to mental health problems among seafarers at individual and organization levels. They include promoting health behaviors, developing resilience, and managing obesity and chronic diseases at the individual level. At the organization level, providing seafarers with adequate instrument support, delivering practical support to communicate with customers, managing their distinct work-rest hours and sustaining an adequate effort-reward balance. Further studies are needed in the maritime field such as longitudinal, or experimental studies for empirical evidence.

## Data Availability

Because the study is a scoping review of published studies, the full references of this study have been provided in the references list.

## Abbreviations

BMI: Body Mass Index
JBI: Joanna Briggs Institute
PRISMA-ScR: Preferred Reporting items for Scoping Reviews flow diagram
MBI: Maslach Burnout Inventory
MJSS: Minnesota Job Satisfaction Scale
PSS-4: Perceive stress scale assessment
SDS: Zung Self Rating Depression Scale
CBI: Copenhagen Burnout Inventory
STAI: State-Trait Anxiety Inventory
